# Left ventricular remodeling, mechanics, and the COAPT trial

**DOI:** 10.3389/fcvm.2023.1124727

**Published:** 2023-01-31

**Authors:** Christos G. Mihos

**Affiliations:** Echocardiography Laboratory, Division of Cardiology, Columbia University Irving Medical Center, Mount Sinai Heart Institute, Miami Beach, FL, United States

**Keywords:** echocardiography, edge-to-edge repair, global longitudinal strain (GLS), left ventricular remodeling, mitral valve repair (MV repair), secondary mitral regurgitation (SMR), speckle-tracking echocardiography

##  1. Introduction

Symptomatic mitral valve (MV) regurgitation is a common cause of morbidity amongst cardiovascular disease patients, with a 1.3-fold increase in prevalence per decade of life past 60 years of age (1). More specifically, secondary mitral regurgitation (SMR) remains a troubling sequela of ischemic heart disease, dilated cardiomyopathy, and atrial myopathy. The mechanisms of SMR are complex, interrelated, and centered on left ventricular (LV) dilatation and abnormal geometry, papillary muscle displacement, MV leaflet tethering, and altered annular shape and mechanics (2, 3). Surgical mitral valve repair (MV repair) has resulted in disappointing long-term outcomes in the SMR population, while replacement predisposes to continued adverse LV remodeling, peri-operative morbidity, and prosthetic valve complications (4, 5).

Conversely, the Cardiovascular Outcomes Assessment of the MitraClip Percutaneous Therapy for Heart Failure Patients with Functional Mitral Regurgitation (COAPT) trial showed that in carefully selected patients with moderate-to-severe SMR, MV transcatheter edge-to-edge repair (TEER) improves heart failure symptoms and survival, and decreases heart failure hospitalizations at 2-year follow-up when compared with medical therapy alone (6). In a recent post-hoc analysis the COAPT investigators analyzed risk of death or heart failure hospitalization according to baseline LV global longitudinal strain (GLS), which is a robust, reproducible, and sensitive marker of myocardial function. Between 10 and 24 months of follow-up, patients with a GLS <−13.2% had a lower risk of death or heart failure hospitalization when compared with those having a GLS >−10.8%, although all patients receiving TEER garnered clinical benefit when compared with medical therapy alone (7). Herein we discuss the importance of LV remodeling and mechanics assessed by 2D and speckle-tracking echocardiography in patients with cardiomyopathy, and its application in the SMR and TEER population.

##  2. Left ventricular deformation, shape, and mechanics

**FIGURE 1:**
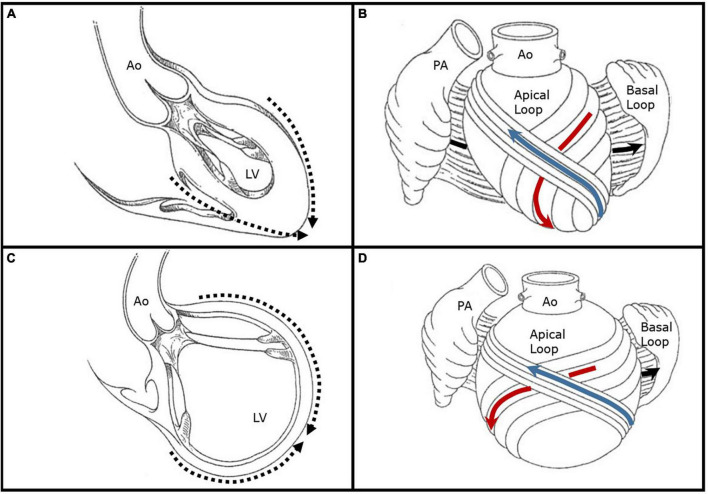
Illustrative representations of left ventricular remodeling and alteration in chamber shape and myofiber orientation. **(A)** Depicted is a left ventricle with a normal elliptical shape forming an apical cap (dashed arrows) with preserved papillary muscle orientation. **(B)** The basal ventricular loop is depicted open with circumferential fibers in a transverse orientation (black arrow) which surround an inner apical loop helix. The descending limb of the apical loop (red arrow) converges into a vortex at the apical cap which then crosses superficially to form the ascending limb (blue arrow). The myofibers of the apical loop limbs are oriented obliquely at approximately 60° angles, and their normal shortening of 15% produces an ejection fraction of 60%. **(C)** Depicted is a remodeled left ventricle with a spherical shape and lack of a true apex (dashed arrows). The chamber is markedly dilated and there is papillary muscle displacement. **(D)** In the setting of left ventricular dilatation and sphericity, the apical loop myofibers are stretched and adapt a transverse orientation similar to the basal loop. Transverse, or circumferential, fiber shortening generates an LV ejection fraction closer to 30%. Figures are adapted with permission from Buckberg et al. (19). Ao, aorta; LV, left ventricle; PA, pulmonary artery.

The ventricular myocardium is composed of a surrounding basal loop with circumferential fibers in a transverse orientation, and an inner apical loop helix with oblique fibers oriented at approximately 60° angles (8). The interaction between the basal and apical loops results in systolic ejection, diastolic relaxation, and ventricular torsion. Essential to proper mechanics is the elliptical shape of the LV, which supports the oblique orientation of the apical loop limbs. In this state, a normal 15% myofiber shortening results in a LV ejection fraction of 60% through efficient myocardial contraction, shortening, and dispersed shearing forces. Conversely, in the setting of LV dilatation and sphericity, the myofibers are stretched and adapt a transverse orientation similar to the basal loop; transverse, or circumferential, fiber shortening generates an LV ejection fraction closer to 30% (9). This latter LV remodeling provides the substrate for papillary muscle displacement, incomplete MV systolic closure, and SMR (Figure 1).

The electromechanical activation of the apical loop results in longitudinal ventricular shortening, which can be assessed by measuring GLS using speckle-tracking echocardiography. Importantly, full wall GLS takes into account the pivotal transmural mechanical interaction between the subendocardial, mid-wall, and subepicardial myofibers (10). GLS is useful in detecting subclinical myocardial dysfunction in the setting of preserved LVEF, while in dilated and ischemic cardiomyopathy it can provide insight into the degree of myocardial impairment (11). In a large study by Namazi et al. (12) of 650 patients with moderate or greater SMR, a GLS >−7% was independently associated with increased all-cause mortality (HR 1.34, 95% CI 1.04–1.72, *p* = 0.02) at a median follow-up of 56 months. When compared with patients who had a GLS <−7%, those with GLS >−7% had more extensive LV remodeling with larger end-diastolic (124 vs. 92 ml/m^2^, *p* < 0.001) and end-systolic volume (96 vs. 63 ml/m^2^, *p* < 0.001) indices, and higher filling pressures (12).

## 3. The COAPT study and LV remodeling

The impact of LV remodeling on the performance of TEER is critical in interpreting the results of the COAPT study, and in translating its success to clinical practice. The inclusion criteria for enrollment in COAPT were: (1) symptomatic heart failure and moderate to severe SMR despite optimal guideline-directed medical therapy; (2) LVEF 20–50%; (3) LV end-systolic diameter <70 mm; (4) pulmonary artery systolic pressure <70 mmHg; and, (5) no evidence of significant right ventricular dysfunction. The 614 patients enrolled in COAPT had a mean LV end-diastolic volume index, LVEF, and GLS of 101 ml/m^2^, 31%, and −11.9%, respectively. These characteristics are in sharp contrast to the Multicentre Study of Percutaneous Mitral Valve Repair MitraClip Device in Patients with Severe Secondary Mitral Regurgitation (MITRA-FR) trial, which showed no benefit of TEER in 307 patients randomized to percutaneous MR correction vs. medical therapy (13). The main inclusion criteria for MITRA-FR were: (1) symptomatic heart failure and severe SMR with guideline-directed medical therapy per “real-world” practice; and, (2) LVEF 15–40%. The mean LV end-diastolic volume index and LVEF in MITRA-FR were 135 ml/m^2^ and 33%, with no data on GLS reported.

It is hypothesized that the discrepancy between COAPT and MITRA-FR regarding the benefits of TEER in patients with SMR was influenced by the extent of LV remodeling and alteration of mechanics. An important caveat to consider when interpreting the study results and emphasizing LV remodeling is that different echocardiography core labs were utilized by the respective trialists, which could potentially introduce uncontrollable confounding. Nevertheless, the patients in COAPT were reported to have a significantly smaller LV size and had to meet a specific cut-off for inclusion based on the maximum systolic chamber dimension, despite a similar LVEF when compared with MITRA-FR patients. It is well established in the surgical literature that the extent of pre-operative LV remodeling is one of the most powerful predictors of a durable MV repair and that there is a threshold beyond which the LV damage and fibrosis is irreversible (14). Important echocardiographic LV parameters that predict MV repair failure include an LV end-diastolic diameter >65 mm or a systolic sphericity index ≥0.7 (15, 16). GLS has been shown to perform well as surrogate marker for LV replacement fibrosis detected by cardiac magnetic resonance imaging in patients with dilated cardiomyopathy, with a value of >−7.9% having an area under the curve of 0.74 (*p* < 0.05) (17). In patients undergoing TEER, a baseline GLS >−9.3% predicts lack of LV reverse remodeling and persistent LV dilatation and sphericity at 2-year follow-up (AUC 0.84, *p* < 0.001) (18). A fair interpretation of this data within the context of the GLS observations from COAPT and the study by Namazi et al. (12), is that similar to patients undergoing surgical MV repair, TEER in the setting of advanced LV remodeling and fibrosis provides minimal clinical benefit at the expense of procedural risk and healthcare-related costs.

## 4. Conclusion

While the aforementioned hypothesis regarding LV remodeling and GLS in patients with SMR undergoing TEER requires prospective and external validation, it provides important insights into appropriate patient selection and factors contributing to the success and application of this relatively new therapy. Pure SMR is primarily a ventricular disease with concomitant histopathologic mitral leaflet remodeling, and as such, it is advisable that our inclusion criteria when selecting candidates for TEER be modeled strictly after those applied in the COAPT trial. Significant GLS impairment may be considered as a risk factor for a lack of LV reverse remodeling and suboptimal outcomes after TEER, with specific cut-off values for clinical use to be determined. Additionally, maximally tolerated guideline-directed medical therapy, as well as cardiac resynchronization therapy when appropriate, are of paramount importance. Taking these factors into account may allow for appropriate risk stratification, expectant management, and a pathway to improved patient outcomes.

## Author contributions

The author confirms being the sole contributor of this work and has approved it for publication.
